# Pragmatic Hypotheses in the Evolution of Science

**DOI:** 10.3390/e21090883

**Published:** 2019-09-11

**Authors:** Luis Gustavo Esteves, Rafael Izbicki, Julio Michael Stern, Rafael Bassi Stern

**Affiliations:** 1 Instituto de Matemática e Estatística, Universidade de São Paulo, São Paulo 05508-090, Brazil; lgesteves2011@gmail.com; 2 Departamento de Estatística, Universidade Federal de São Carlos, São Paulo 13565-905, Brazil; rafaelizbicki@gmail.com (R.I.); rbstern@gmail.com (R.B.S.)

**Keywords:** hypothesis tests, precise hypotheses, pragmatic hypotheses

## Abstract

This paper introduces pragmatic hypotheses and relates this concept to the spiral of scientific evolution. Previous works determined a characterization of logically consistent statistical hypothesis tests and showed that the modal operators obtained from this test can be represented in the hexagon of oppositions. However, despite the importance of precise hypothesis in science, they cannot be accepted by logically consistent tests. Here, we show that this dilemma can be overcome by the use of pragmatic versions of precise hypotheses. These pragmatic versions allow a level of imprecision in the hypothesis that is small relative to other experimental conditions. The introduction of pragmatic hypotheses allows the evolution of scientific theories based on statistical hypothesis testing to be interpreted using the narratological structure of hexagonal spirals, as defined by Pierre Gallais.

## 1. Introduction

Standard hypothesis tests can lead to immediate logical incoherence, which makes their conclusions hard to interpret. This incoherence occurs because such tests have only two possible outcomes. Indeed, Izbicki and Esteves [1] shows that there exists no two-valued test that satisfies desirable statistical properties and is also logically coherent.

**Figure 1:**
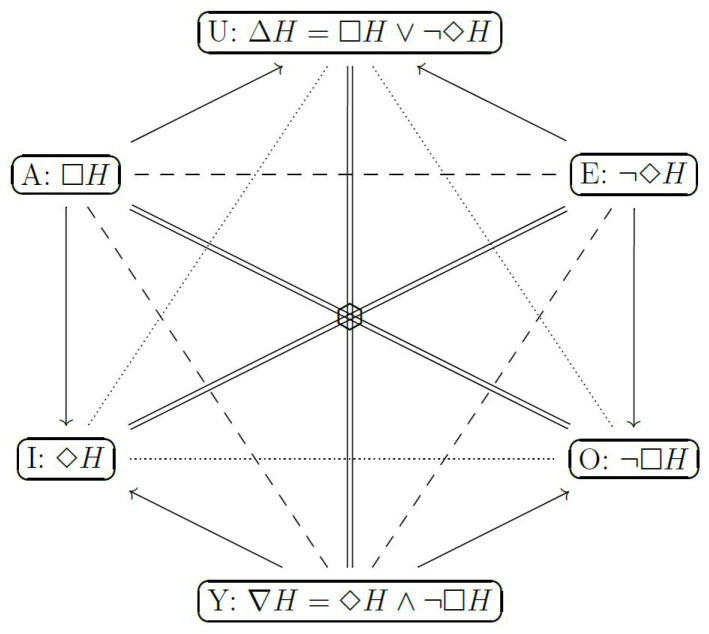
Hexagons of opposition for statistical modalities.

In order to overcome such an impossibility result, Esteves et al. [2] propose agnostic hypothesis tests, which have three possible outputs: (A) accept the hypothesis, say *H*, (E) reject *H*, or (Y) remain agnostic about *H*. These tests can be made logically coherent while preserving desirable statistical properties. For instance, both conditions are satisfied by the Generalized Full Bayesian Significance Test (GFBST). Furthermore, Stern et al. [3] show that the GFBST’s modal operators and their respective negations can be represented by vertices of the hexagon of oppositions [4,5,6,7,8,9], which is depicted in Figure 1.

**Figure 2:**
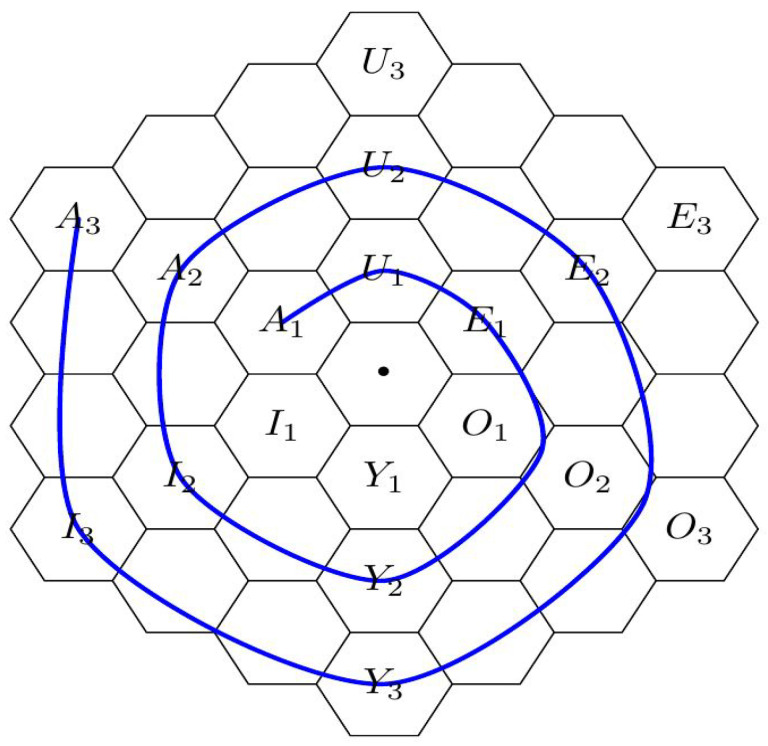
Gallais’ evolutionary spiral.

This paper complements the above static representation with an analysis of the GFBST in the dynamic evolution of scientific theories. The analysis is based on the metaphor of evolutive hexagonal spirals [10,11], in which the logical modalities associated to scientific theories change over time, as in Figure 2. Our key point in this paradigm is reconciling two apparently contradictory facts. On the one hand, precise or sharp hypotheses, that is, hypotheses that have a priori zero probability are central in scientific theories [12,13]. On the other hand, the GFBST never accepts (A) precise hypotheses. These observations lead to the apparent paradox that, if the GFBST were used to test scientific theories, then the acceptance step in the spiral of scientific theories would be forfeited.

We overcome this paradox by proposing the concept of a “pragmatic hypothesis” associated to a precise hypothesis. Although precise hypotheses are commonly obtained from mathematical theories used in areas of science and technology [12,13], the associated pragmatic hypothesis is an imprecise hypothesis that is sufficiently good from the practical purpose of an end-user of the theories. For instance, Newtonian theory assumes a gravitational force of magnitude given by the equation $F=G\;m_{1}\;m_{2}\;d^{-2}$, where the gravitational constant *G* has a precise value. However, the current Committee on Data for Science and Technology (CODATA) value for the gravitational constant is $G=6.67408\left(31\right)\times 10^{-11}m^{3}\;{\mathrm{kg}}^{-1}\;s^{-2}$, which includes a standard deviation for the last significant digits, 408±31. Thus, it may be reasonable for a given end-user to assume that the theoretical form of the last equation is exact, but that, pragmatically, the constant *G* can only be known up to a chosen precision. As a result, one might wish to test an imprecise hypothesis associated to the scientific hypothesis of interest [14,15].

This article advocates for the conceptual distinction between a precise scientific theory and an associated pragmatic hypotheses. The alternate use of precise and pragmatic versions of corresponding statistical hypotheses enables the GFBST to (pragmatically) accept scientific hypotheses. Moreover, this alternate use allows the GFBST to track the evolution of scientific theories, as interpreted in the context of Gallais’ hexagonal spirals.

Our main goal in this paper is to formalize testing procedures for a theory taking into consideration the level of precision that is appropriate for a given end-user. To handle this problem, we consider the end-user’s predictions about an experiment of his interest. The variation in these predictions can be explained by a combination of the level of imprecision in the theory and by properties of the end-user’s experiment. For instance, the latter source of variation is influenced by properties of the equipment, including the precision, accuracy, and resolution of measuring devices [16,17], and also error bounds for fundamental constants and calibration factors [18,19,20,21,22,23,24,25,26]. We propose to choose a pragmatic hypothesis in such a way that the imprecision in the end-user’s predictions is mostly due to his experimental conditions and not due to the level of imprecision in the theory that he uses.

In order to develop this argument, Section 2 first adapts Gallais’s metaphor of hexagonal spirals to the evolution of science. Next, Section 3 proposes three methods of decomposing the variability in an end-user’s predictions into the level of precision of the theory he uses and his experimental conditions. Section 3.1 and Section 3.2 use these decompositions in order to build pragmatic hypothesis. They build pragmatic hypotheses for simple hypotheses and then prove that there exists a single way of extending this construction to composite hypotheses while preserving logical coherence in simultaneous hypothesis testing. This methodology is illustrated in Section 4. All proofs are found in Appendix A.

## 2. Gallais’ Hexagonal Spirals and the Evolution of Science

Following a well-established tradition in structural semantics and narratology [27,28], Gallais and Pollina [10] propose that many classical medieval tales follow the same organizational pattern. More precisely, these narratives exhibit an underlying “intellectual structure” and are organized according to an underlying archetypal format or prototypical pattern. This pattern includes both static and a dynamical aspects. From a static perspective, the logical structure of the narrative is such that each arch is represented by a vertex of the “hexagon of oppositions” [4]. The static hexagon of oppositions is depicted in Figure 1 and represents in each vertex a modal operator among necessity (□), possibility (⋄), contingency (Δ), and their negations (¬). These modal operators are structured according to three axes of opposition, (===); a triangle of contrariety, (−−−); another triangle of sub-contrariety, (⋯); and several edges of subalteration (⟶). From a dynamical perspective, the temporal evolution of the narrative follows a spiral (Figure 2) that unwinds (se déroule) around concentric and expanding hexagons of opposition [10,11].

Because the evolution of science can also be conceived as following a spiral pattern [29], its analysis can benefit from the structure in the works by the authors of [10,11]. From a static perspective, the logical modalities induced by agnostic hypothesis tests [3] can be represented in the hexagon of oppositions. From a dynamic perspective, scientific theories evolve as a spiral which unwinds around the following states:

- $A_{1}$- Extant thesis: This vertex represents a standing paradigm, an accepted theory using well-known formalisms and familiar concepts, relying on accredited experimental means and methods, etc. In fact, the concepts of a current paradigm may become so familiar and look so natural that they become part of a reified ontology. That is, there is a perceived correspondence between concepts of the theory and “dinge-an-sich” (things-in-themselves) as seen in nature [29,30].
- $U_{1}$- Analysis: This vertex represents the moment when some hypotheses of the standing theory are put in question. At this moment, possible alternatives to the standing hypotheses may still be only vaguely defined.
- $E_{1}$- Antithesis: This vertex represents the moment when some laws of the standing theory have to be rejected. Such a rejection of old laws may put in question the entire world-view of the current paradigm, opening the way for revolutionary ideas, as described in the next vertex.
- $O_{2}$- Apothesis/ Prosthesis: This vertex is the locus of revolutionary freedom. Alternative models are considered, and specific (precise) forms investigated. There is intellectual freedom to set aside and dispose of (apothesis) old preconceptions, prejudices and stereotypes, and also to explore and investigate new paths, to put together (prosthesis) and try out new concepts and ideas.
- $Y_{2}$- Synthesis: It is at this vertex that new laws are formulated; this is the point of Eureka moment(s). A selection of old and and new concepts seem to click into place, fitting together in the form of new laws, laws that are able to explain new phenomena and incorporate objects of an expanded reality.
- $I_{2}$- Enthesis: At this vertex new laws, concepts and methods must enter and be integrated into a consistent and coherent system. At this stage many tasks are performed in order to combine novel and traditional pieces or to accommodate original and conventional components into an well-integrated framework. Finally, new experimental means and methods are developed and perfected, allowing the new laws to be corroborated.
- $A_{2}$- New Thesis: At this vertex, the new theory is accepted as the standard paradigm that succeeds the preceding one ($A_{1}$). Acceptance occurs after careful determination of fundamental constants and calibration factors (including their known precision), metrological and instrumentational error bounds, etc. At later stages of maturity, equivalent theoretical frameworks may be developed using alternative formalisms and ontologies. For example, analytical mechanics offers variational alternatives that are (almost) equivalent to the classical formulation of Newtonian mechanics [31]. Usually, these alternative worldviews reinforce the trust and confidence on the underlying laws. Nevertheless, the existence of such alternative perspectives may also foster exploratory efforts and investigative works in the next cycle in evolution.

Table 1 applies this spiral structure to the evolution of the theories of orbital astronomy and chemical affinity. The evolution of orbital astronomy has been widely studied [32]. The evolution of chemical affinity is presented in greater detail in Stern [29], Stern and Nakano [33].

The above spiral structure highlights that a statistical methodology should be able to obtain each of the six modalities in the hexagon of oppositions. Before an acceptance vertex (A) in the hexagon is reached by the spiral of scientific evolution, theoretically precise or sharp hypotheses must be formulated. However, a logically coherent hypothesis test, such as the GFBST, can choose solely between rejecting or remaining agnostic (i.e., corroborating) such sharp hypotheses. Once the evolving theory becomes (part of) a well-established paradigm, the GFBST can be used with the goal of accepting non-sharp hypotheses in the context of the same paradigm, a context that includes fundamental constants and calibration factors (and their respective uncertainties), metrological error bounds, specified accuracies of scientific intrumentation, etc. The non-sharp versions of sharp hypotheses used in such tests are called pragmatic, and their formulation is developed in the following sections.

## 3. Pragmatic Hypotheses

In order to derive pragmatic hypotheses from precise ones, it is necessary to define an idealized future experiment. Let θ be an unknown parameter of interest, which is used to express scientific hypotheses and that takes values in the parameter space, Θ. A scientific hypothesis takes the form $H_{0}:\theta \in \Theta_{0}$, where $\Theta_{0}\subset \Theta$. Whenever there is no ambiguity, $H_{0}$ and $\Theta_{0}$ are used interchangeably. Also, the determination of θ is useful for predicting an idealized future experiment, Z, which takes values in Z. The uncertainty about *Z* depends on θ by means of $P_{\theta^{*}}$, the probability measure over Z when it is known that $\theta =\theta^{*}$, $\theta^{*}\in \Theta$.

Often, it is sufficient for an end-user to determine a pragmatic hypothesis, that is, that the parameter lies in a set of plausible values, which is larger than the null hypothesis. This set can be chosen in such a way that the variation over predictions about a future experiment is mostly due to experimental conditions rather than to the imprecision in the value of the parameter. This section formally develops a methodology for determining these pragmatic hypotheses.

In order to compare two parameter values, we use a “predictive dissimilarity”, $d_{Z}$, which is a function, $d_{Z}:\Theta \times \Theta \to R^{+}$, such that $d_{Z}\left(\theta_{0},\theta^{*}\right)$ measures how much the predictions made for Z based on $\theta^{*}$ diverge from the ones made based on $\theta_{0}$. We define and compare three possible choices for such a dissimilarity.

**Definition** **1.** *The Kullback–Leibler predictive dissimilarity, ${KL}_{Z}$, is*

$$\begin{array}{cc} {KL}_{Z}\left(\theta_{0},\theta^{*}\right)\; & =KL\left(P_{\theta^{*}},P_{\theta_{0}}\right)=\int_{Z}\log\left(\frac{dP_{\theta^{*}}}{dP_{\theta_{0}}}\right)dP_{\theta^{*}}, \end{array}$$

that is, ${KL}_{Z}\left(\theta_{0},\theta^{*}\right)$ is the relative entropy between $P_{\theta^{*}}$ and $P_{\theta_{0}}$.

**Example** **1** (Gaussian with known variance.)**.** *Let $Z=\left(Z_{1}\ldots ,Z_{d}\right)∼N\left(\theta ,\Sigma_{0}\right)$ be a random vector with a multivariate Gaussian distribution:*

$$\begin{array}{cc} \frac{dP_{\theta }\left(z\right)}{dz}\; & =∥2\pi \Sigma_{0}{∥}^{-0.5}\exp\left(-0.5{\left(z-\theta \right)}^{t}\Sigma_{0}^{-1}d\left(z-\theta \right)\right)\\ {KL}_{Z}\left(\theta_{0},\theta^{*}\right)\; & =\int_{R^{d}}\log\left(\frac{dP_{\theta^{*}}\left(z\right)}{dP_{\theta_{0}}\left(z\right)}\right)dP_{\theta^{*}}\left(z\right)=0.5{\left(\theta_{0}-\theta^{*}\right)}^{t}\Sigma_{0}^{-1}\left(\theta_{0}-\theta^{*}\right), \end{array}$$

*When d=1 and $\Sigma_{0}=\sigma_{0}^{2}$,*

(1) $$\begin{array}{cc} KL_{z}\left(\theta_{0},\theta^{*}\right)\; & =\frac{{\left(\theta_{0}-\theta^{*}\right)}^{2}}{2\sigma_{0}^{2}} \end{array}$$

The KL dissimilarity evaluates the distance between the predictive probability distributions for the future experiment under two parameter values, $\theta_{0}$ and $\theta^{*}$. Although the KL dissimilarity is general, it can be challenging to interpret. In particular, it can be hard to establish the quality of the predictions for Z based on $\theta^{*}$ when Z is actually generated from $\theta_{0}$ and $KL_{Z}\left(\theta_{0},\theta^{*}\right)\leq \epsilon$. A more interpretable dissimilarity is obtained by taking $d_{Z}\left(\theta_{0},\theta^{*}\right)$ to measure how far are the best predictions for Z based on $\theta^{*}$ and $\theta_{0}$. In this case, if one makes a prediction for Z based on $\theta^{*}$, $z_{*}$, and Z was actually generated using $\theta_{0}$, then $d_{Z}\left(\theta_{0},\theta^{*}\right)\leq \epsilon$ guarantees that $z_{*}$ will be at most ϵ apart from the best possible prediction. Such a dissimilarity is discussed in the following definition.

**Definition** **2** (Best prediction dissimilarity—BP.)**.** *Let $\hat{Z}:\Theta \to Z$ be such that $\hat{Z}\left(\theta_{0}\right)$ is the best prediction for Z given that $\theta =\theta_{0}$. For example, one can take*

$$\begin{array}{cc} \hat{Z}\left(\theta_{0}\right)\; & =\arg\min_{z\in Z}\delta_{Z,\theta_{0}}\left(z\right), \end{array}$$

where $\delta_{Z,\theta_{0}}:Z\to R$ is such that $\delta_{Z,\theta_{0}}\left(z\right)$ measures how bad z predicts Z when $\theta =\theta_{0}$. The “best prediction dissimilarity”, ${BP}_{Z}\left(\theta_{0},\theta^{*}\right)$, measures how badly $\hat{Z}\left(\theta^{*}\right)$ predicts Z relatively to $\hat{Z}\left(\theta_{0}\right)$ when $\theta =\theta_{0}$. Formally,

$$\begin{array}{cc} {BP}_{Z}\left(\theta_{0},\theta^{*}\right)\; & =g\left(\frac{\delta_{Z,\theta_{0}}\left(\hat{Z}\left(\theta^{*}\right)\right)-\delta_{Z,\theta_{0}}\left(\hat{Z}\left(\theta_{0}\right)\right)}{\delta_{Z,\theta_{0}}\left(\hat{Z}\left(\theta_{0}\right)\right)}\right), \end{array}$$

*where g:R⟶R is a motononic function. The choice of g in a particular setting aims at improving the interpretation of the best prediction dissimilarity criterion.*

**Example** **2** (BP under quadratic form.)**.** *Let $Z=R^{d}$, $\mu_{Z,\theta }=E\left[Z|\theta \right]$, $\Sigma_{Z,\theta }=V\left[Z|\theta \right]$ and S be a positive definite matrix. Define the quadratic form induced by S to be ${∥z∥}_{S}^{2}=z^{T}Sz$ and*

$$\begin{array}{c} \delta_{Z,\theta_{0}}\left(z\right)=E\left[{∥Z-z∥}_{S}^{2}|\theta =\theta_{0}\right] \end{array}$$

The optimal prediction under $\theta^{*}$ is $\hat{Z}\left(\theta^{*}\right)=\mu_{Z,\theta^{*}}$. It follows that

$$\begin{array}{cc} \delta_{Z,\theta_{0}}\left(\hat{Z}\left(\theta^{*}\right)\right)\; & =E\left[∥Z-\mu_{Z,\theta^{*}}{∥}_{S}^{2}|\theta =\theta_{0}\right]\\ \; & =∥\mu_{Z,\theta_{0}}-\mu_{Z,\theta^{*}}{∥}_{S}^{2}+E\left[∥Z-\mu_{Z,\theta_{0}}{∥}_{S}^{2}|\theta =\theta_{0}\right] \end{array}$$

*In particular, $\delta_{Z,\theta_{0}}\left(\hat{Z}\left(\theta_{0}\right)\right)=E\left[∥Z-\mu_{Z,\theta_{0}}{∥}_{S}^{2}|\theta =\theta_{0}\right]$. Therefore,*

(2) $$\begin{array}{cc} {BP}_{Z}\left(\theta_{0},\theta^{*}\right)\; & =g\left(\frac{∥\mu_{Z,\theta_{0}}-\mu_{Z,\theta^{*}}{∥}_{S}^{2}}{E\left[∥Z-\mu_{Z,\theta_{0}}{∥}_{S}^{2}|\theta =\theta_{0}\right]}\right) \end{array}$$

*In this example, BP${}_{Z}$ can be put in the same scale as Z by taking $g\left(x\right)=\sqrt{x}$. Also, two choices of S are of particular interest. When $S=V{\left[Z|\theta =\theta_{0}\right]}^{-1}$, Equation (2) simplifies to*

(3) $$\begin{array}{cc} {BP}_{Z}\left(\theta_{0},\theta^{*}\right)\; & =g\left(d^{-1}{∥\mu_{Z,\theta_{0}}-\mu_{Z,\theta^{*}}∥}_{\Sigma_{Z,\theta_{0}}^{-1}}^{2}\right) \end{array}$$

*Similarly, when S is the identity matrix, Equation (2) simplifies to*

(4) $$\begin{array}{c} {BP}_{Z}\left(\theta_{0},\theta^{*}\right)=g\left(\frac{∥E\left[Z|\theta =\theta_{0}\right]-E\left[Z|\theta =\theta^{*}\right]{∥}_{2}^{2}}{tr(V\left[Z|\theta =\theta_{0}\right])}\right) \end{array}$$

Equation (4) admits an intuitive interpretation. The larger the value of $tr(V\left[Z|\theta =\theta_{0}\right])$, the more Z is dispersed and the harder it is to predict its value. Also, $∥E\left[Z|\theta =\theta_{0}\right]-E\left[Z|\theta =\theta^{*}\right]{∥}_{2}^{2}$ measures how far apart are the best prediction for Z under $\theta =\theta_{0}$ and $\theta =\theta^{*}$. That is, ${\mathrm{BP}}_{Z}\left(\theta_{0},\theta^{*}\right)$ captures that, if one predicts Z assuming that $\theta =\theta^{*}$ when it is actually $\theta =\theta_{0}$, then the error with respect to the best prediction is increased as a function of the distance between the predictions over the dispersion of Z.

**Example** **3** (Gaussian with known variance.)**.** *Consider Example 1 and let $\delta_{Z,\theta_{0}}\left(z\right)$ be as in Example 2. It follows from Equation (4) that when S is the identity matrix,*

(5) $$\begin{array}{cc} {BP}_{Z}\left(\theta_{0},\theta^{*}\right)\; & =g\left(\frac{∥\theta_{0}-\theta^{*}{∥}_{2}^{2}}{tr(\Sigma_{0})}\right) \end{array}$$

*Similarly, it follows from Equation (3) that when $S=\Sigma_{0}^{-1}$,*

(6) $$\begin{array}{cc} {BP}_{Z}\left(\theta_{0},\theta^{*}\right)\; & =g\left(d^{-1}{\left(\theta_{0}-\theta^{*}\right)}^{t}\Sigma_{0}^{-1}\left(\theta_{0}-\theta^{*}\right)\right) \end{array}$$

Conclude from Equation (6) that, if $S=\Sigma_{0}^{-1}$ and g(x)=x, then ${BP}_{Z}\left(\theta_{0},\theta^{*}\right)=2d^{-1}{KL}_{Z}\left(\theta_{0},\theta^{*}\right)$. Also, when d=1, $\Sigma_{0}=\sigma_{0}^{2}$ and $g\left(x\right)=\sqrt{x}$, both Equations (5) and (6) simplify to

(7) $$\begin{array}{cc} {BP}_{Z}\left(\theta_{0},\theta^{*}\right)\; & =\sigma_{0}^{-1}\left|\theta_{0}-\theta^{*}\right| \end{array}$$

In some situations, Z is the average of m independent observations distributed as $N(\theta ,\Sigma_{0})$. In this case, $Z∼N(\theta ,m^{-1}\Sigma_{0})$. It follows from Equation (5) that $BP_{Z}\left(\theta_{0},\theta^{*}\right)=g\left(\frac{m∥\theta_{0}-\theta^{*}{∥}_{2}^{2}}{tr(\Sigma_{0})}\right)$ when S is the identity, and ${BP}_{Z}\left(\theta_{0},\theta^{*}\right)=g\left(md^{-1}{\left(\theta_{0}-\theta^{*}\right)}^{t}\Sigma_{0}^{-1}\left(\theta_{0}-\theta^{*}\right)\right)$ when $S=\Sigma_{0}^{-1}$.

Although ${\mathrm{BP}}_{Z}$ is more interpretable then ${\mathrm{KL}}_{Z}$, it also relies on more tuning variables, such as δ, $\hat{Z}$, and *g*. A balance between these features is obtained by a third predictive dissimilarity, which evaluates how easy it is to recover the value of θ between $\theta_{0}$ or $\theta^{*}$ based on Z.

**Definition** **3** (Classification distance—CD.)**.** Let ${\hat{\theta }}_{\theta_{0},\theta^{*}}:Z\to \Theta$ be such that

$$\begin{array}{cc} {\hat{\theta }}_{\theta_{0},\theta^{*}}\left(z\right) & =\arg\max_{\theta \in \{\theta_{0},\theta^{*}\}}f_{Z}\left(z|\theta \right) \end{array}$$

${\hat{\theta }}_{\theta_{0},\theta^{*}}$ assigns to each possible outcome of the future experiment z, in which the values of θ, $\theta_{0}$, or $\theta^{*}$ make the experimental result more likely. The classification distance between $\theta_{0}$, $\theta^{*}$, and $CD(\theta_{0},\theta^{*})$ is defined as

$$\begin{array}{cc} CD(\theta_{0},\theta^{*})\; & =0.5P\left({\hat{\theta }}_{\theta_{0},\theta^{*}}\left(Z\right)=\theta_{0}|\theta_{0}\right)+0.5P\left({\hat{\theta }}_{\theta_{0},\theta^{*}}\left(Z\right)=\theta^{*}|\theta^{*}\right)-0.5 \end{array}$$

$CD(\theta_{0},\theta^{*})+0.5$ is the best Bayes utility in an hypothesis test of $\theta_{0}$ against $\theta^{*}$ using a uniform prior for θ and the 0/1 utility [15]. By subtracting 0.5 from this quantity, $CD(\theta_{0},\theta^{*})$ varies between 0 and 0.5 and is a distance. Also,

$$\begin{array}{cc} CD(\theta_{0},\theta^{*})\; & =0.5TV\left(P_{\theta_{0}},P_{\theta^{*}}\right)=0.25{∥P_{\theta_{0}}-P_{\theta^{*}}∥}_{1}, \end{array}$$

where $TV\left(P_{\theta_{0}},P_{\theta^{*}}\right)=\sup_{A}\left|P_{\theta_{0}}\left(A\right)-P_{\theta^{*}}\left(A\right)\right|$ and $∥P_{\theta_{0}}-P_{\theta^{*}}{∥}_{1}=\int_{Z}|P_{\theta_{0}}\left(z\right)-P_{\theta^{*}}\left(z\right)|dz$ is the $L_{1}$-distance between probability measures.

**Example** **4** (Gaussian with known variance.)**.** *Consider Examples 1 and 3, when d=1, $\Sigma_{0}=\sigma_{0}^{2}$, obtain*

(8) $$\begin{array}{cc} {CD}_{Z}\left(\theta_{0},\theta^{*}\right)\; & =\Phi \left(\frac{|\theta_{0}-\theta^{*}|}{2\sigma_{0}}\right)-\frac{1}{2} \end{array}$$

*Note that, in this case, CD would be the same as BP if, instead of taking $g\left(x\right)=\sqrt{x}$, one chose $g\left(x\right)=\Phi \left(0.5\sqrt{x}\right)-0.5$.*

Although analytical expressions for CD are generally not available, it is possible to approximate it via numerical integration methods.

The choice between predictive dissimilarity functions depends on the type of guarantee the end-user wishes to obtain. In particular, BP, KL, and CD is not an exhaustive list of dissimilarities. However, some of their properties can be useful in obtaining a choice. For instance, although BP yields a metric on the parameter space, KL and CD are dissimilarities between probability functions. That is, although BP will generate pragmatic hypothesis that have parameter values numerically close to a given $\theta_{0}$, KL and CD will yield pragmatic hypothesis that have parameter values lead to similar prediction about Z. Also, although KL evaluates similarity between predictions from an information theoretic perspective, CD evaluates them from a perspective of hypothesis tests.

### 3.1. Singleton Hypotheses

We start by defining the pragmatic hypothesis associated to a singleton hypothesis. A singleton hypothesis is one in which the parameter assumes a single value, such as $H_{0}:\theta =\theta_{0}$. In this case, the pragmatic hypothesis associated to $H_{0}$ is the set of points whose dissimilarity to $\theta_{0}$ is at most ϵ, as formalized below.

**Definition** **4** (Pragmatic hypothesis for a singleton.)**.** *Let $H_{0}:\theta =\theta_{0}$, $d_{Z}$ be a predictive dissimilarity function and ϵ>0. The pragmatic hypothesis for $H_{0}$, $Pg(\left\{\theta_{0}\right\},d_{Z},\epsilon )$, is*

$$\begin{array}{cc} Pg(\left\{\theta_{0}\right\},d_{Z},\epsilon )\; & =\{\theta^{*}\in \Theta :d_{Z}\left(\theta_{0},\theta^{*}\right)\leq \epsilon \} \end{array}$$

Note that for $\epsilon_{1}<\epsilon_{2}$, $Pg\left(\left\{\theta_{0}\right\},d_{Z},\epsilon_{1}\right)\subseteq Pg\left(\left\{\theta_{0}\right\},d_{Z},\epsilon_{2}\right)$.

**Example** **5** (Gaussian with known variance)**.** *Consider Examples 1 and 3 when d=1, $\Sigma_{0}=\sigma_{0}^{2}$ and $g\left(x\right)=\sqrt{x}$. It follows from Equations (1), (7) and (8) that*

$$\begin{array}{cc} Pg(\left\{\theta_{0}\right\},BP_{Z},\epsilon )\; & =\left[\theta_{0}-\epsilon \sigma_{0},\theta_{0}+\epsilon \sigma_{0}\right]\\ Pg(\left\{\theta_{0}\right\},KL_{Z},\epsilon )\; & =\left[\theta_{0}-\sqrt{2\epsilon }\sigma_{0},\theta_{0}+\sqrt{2\epsilon }\sigma_{0}\right]\\ Pg(\left\{\theta_{0}\right\},CD_{Z},\epsilon )\; & =\left[\theta_{0}-2\Phi^{-1}\left(0.5+\epsilon \right)\sigma_{0},\theta_{0}+2\Phi^{-1}\left(0.5+\epsilon \right)\sigma_{0}\right] \end{array}$$

Note that the size of each of the pragmatic hypothesis is proportional to $\sigma_{0}$. This occurs because each predictive dissimilarity functions makes the prediction error due to the unknown parameter value small with respect to that due to the data variability, $\sigma_{0}^{2}$.

### 3.2. Composite Hypotheses

Next, we consider pragmatic hypotheses for general hypotheses $H_{0}:\theta \in \Theta_{0}$, where $\Theta_{0}\subset \Theta$.

**Definition** **5.** For each hypothesis $\Theta_{0}\subseteq \Theta$, predictive dissimilarity $d_{Z}$ and ϵ>0, $Pg(\Theta_{0},d_{Z},\epsilon )$ is the pragmatic hypothesis associated to $\Theta_{0}$ induced by $d_{Z}$ and ϵ. Whenever $d_{Z}$ and ϵ are clear or not relevant to the result, we write $Pg(\Theta_{0})$ instead of $Pg(\Theta_{0},d_{Z},\epsilon )$.

**Figure 3:**
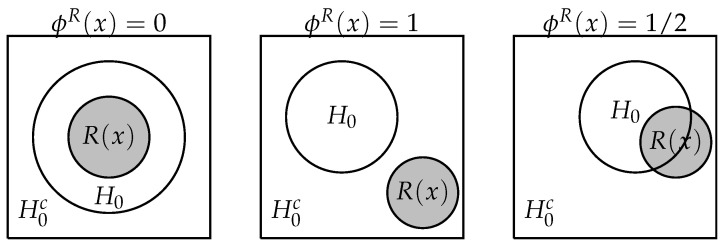
ϕ(x) is an agnostic test based on the region estimator R(x) for testing $H_{0}$.

In order to construct these pragmatic hypotheses, we use logically coherent agnostic hypothesis tests. For each hypothesis, an agnostic hypothesis test can either reject it (1), accept it (0), or remain agnostic (1/2) [34]. Esteves et al. [2] shows that an agnostic hypothesis test is logically coherent if and only if it is based on a region estimator. Such tests are presented in Definition 7 and illustrated in Figure 3.

**Definition** **6.** Let X denote the sample space of the data used to test a hypothesis. A region estimator is a function, R:X⟶P(Θ), where P(Θ) is the power set of Θ.

**Definition** **7** (Agnostic test based on a region estimator.)**.** *The agnostic test based on the region estimator R for testing $H_{0}$, $\phi_{H_{0}}^{R}$, such that*

$$\begin{array}{cc} \phi_{H_{0}}^{R}\left(x\right) & =\left\{\begin{array}{cc} 0 & ,\;if\;R\left(x\right)\subseteq H_{0}\\ 1 & ,\;if\;R\left(x\right)\subseteq H_{0}^{c}\\ \frac{1}{2} & ,\;otherwise. \end{array}\right. \end{array}$$

Besides the logical conditions on the hypothesis test, one might also impose logical restraints on how pragmatic hypotheses are constructed. For instance, let *A* and *B* be two hypothesis, such that *B* logically entails *A*, that is, B⊆A. If a logically coherent test accepts *B*, then it also accepts *A*. This property is called monotonocity [1,35,36,37]. One might also impose that Pg, such that if a logically coherent hypothesis test accepts Pg(B), then it should also accept Pg(A). Similarly, let ${\left(A_{i}\right)}_{i\in I}$ be a collection of hypothesis which cover *A*, that is, $A\subseteq \cup_{i\in I}A_{i}$. If a logically coherent hypothesis test rejects every $A_{i}$, then it rejects *A*. This property is called union consonance. One might also impose that Pg is such that, if a logically coherent hypothesis test rejects $Pg(A_{i})$ for every *i*, then it should also reject Pg(A). The above conditions define the logical coherence of a procedure for constructing pragmatic hypotheses.

**Definition** **8.** *A procedure for constructing pragmatic hypothesis, Pg, is logically coherent if, for every logically coherent hypothesis test ϕ and sample point x:*

1. If $\phi_{Pg(B)}\left(x\right)=0$ for some B⊆A, then $\phi_{Pg(A)}\left(x\right)=0$.
2. If $\phi_{Pg(A_{i})}\left(x\right)=1$ for every i∈I and $A\subseteq \cup_{i\in I}A_{i}$, then $\phi_{Pg(A)}\left(x\right)=1$.

In order to motivate the above definition, consider that the frequencies of AA, AB, and BB in a given population are $\theta_{1}$, $\theta_{2}$, and $\theta_{3}$, respectively. Note that B:={0.25,0.5,0.25} is a subset of $A=\{\left(p^{2},2p\left(1-p\right),{\left(1-p\right)}^{2}\right):p\in \left[0,1\right]\}$, which denotes the Hardy–Weinberg equilibrium. That is, if the frequencies AA, AB, and BB are, respectively, 0.25, 0.5, and 0.25, then the population follows the Hardy–Weinberg equilibrium. As a result, if one pragmatically accepts that the population satisfies the specified proportions, then one might also wish to pragmatically accept that the population follows the Hardy–Weinberg. Similarly, if one pragmatically rejects for every p∈[0,1] that the frequencies of AA, AB, and BB are, respectively, $p^{2}$, 2p(1−p), and ${\left(1-p\right)}^{2}$, then one might also wish to pragmatically reject that the population follows the Hardy–Weinberg equilibrium. These conditions are assured in Definition 8.

In a logically coherent procedure for constructing pragmatic hypotheses, the pragmatic hypothesis associated to a composite hypothesis is completely determined by the pragmatic hypotheses associated to simple hypotheses. This result is presented in Theorem 1.

**Theorem** **1.** A procedure for constructing pragmatic hypothesis, Pg, is logically coherent if and only if, for every hypothesis, $\Theta_{0}$, $Pg\left(\Theta_{0}\right)={⋃}_{\theta \in \Theta_{0}}Pg\left(\left\{\theta \right\}\right)$.

Using Theorem 1, it is possible to determine a logically coherent procedure for constructing pragmatic hypotheses by determining only the pragmatic hypothesis associated to simple hypothesis, such as in Section 3.1. Theorem 1 is illustrated in Section 4. One can also obtain the following general relation between predictive dissimilarities.

**Lemma** **1.** $Pg\left(\Theta_{0},KL_{Z},8\epsilon^{2}\right)\subseteq Pg\left(\Theta_{0},CD_{Z},\epsilon \right)$.

Besides being logically coherent, it is often desirable in statistics [38,39] and in science [12,13] for a procedure to be invariant to reparametrization, so as to ensure that the procedure reaches the same conclusions whatever the coordinate system is used to specify both the sample and the parameter spaces. For instance, the pragmatic hypothesis that is obtained using the International metric system should be compatible to the one that is obtained using the English metric system. Invariance to reparametrization is formally presented in Definition 10.

**Definition** **9.** ${\left(P_{\theta^{*}}^{*}\right)}_{\theta^{*}\in \Theta^{*}}$ is a reparameterization of ${\left(P_{\theta }\right)}_{\theta \in \Theta }$ if there exists a bijective function, $f:\Theta \to \Theta^{*}$, such that for every θ∈Θ, $P_{\theta }=P_{f(\theta )}^{*}$.

**Definition** **10.** Let ${\left(P_{\theta^{*}}^{*}\right)}_{\theta^{*}\in \Theta^{*}}$ be a reparametrization of ${\left(P_{\theta }\right)}_{\theta \in \Theta }$ by a bijective function, $f:\Theta \to \Theta^{*}$. Also, let $d_{Z}$ and $d_{Z}^{*}$ be predictive dissimilarity functions. The functions $d_{Z}$ and $d_{Z}^{*}$ are invariant to the reparametrization if for every logically coherent procedure for constructing pragmatic hypotheses, Pg,

$$\begin{array}{cc} f[Pg\left(\Theta_{0},d_{Z},\epsilon \right)]\; & =Pg(f\left[\Theta_{0}\right],d_{Z}^{*},\epsilon ), \end{array}$$

Definition 10 states that, if $\Theta_{0}$ is an hypothesis and invariance to reparametrization holds, then the pragmatic hypothesis obtained in a reparametrization of $\Theta_{0}$, say $Pg(f\left[\Theta_{0}\right])$, is the same as the transformed pragmatic hypothesis associated to $\Theta_{0}$, $f[Pg\left(\Theta_{0}\right)]$. Theorem 2 presents a sufficient condition for obtaining invariance to reparametrization.

**Theorem** **2.** Let ${\left(P_{\theta^{*}}^{*}\right)}_{\theta^{*}\in \Theta^{*}}$ be a reparameterization of ${\left(P_{\theta }\right)}_{\theta \in \Theta }$ given by a bijective function, f. If $d_{Z}$ and $d_{Z}^{*}$ satisfy $d_{Z}\left(\theta_{0},\theta \right)=d_{Z}^{*}\left(f\left(\theta_{0}\right),f\left(\theta \right)\right)$, then $d_{Z}$ and $d_{Z}^{*}$ are invariant to this reparametrization.

**Corollary** **1.** If $d_{Z}$ and $d_{Z}^{*}$ are the same choice between KL, BP, or CD, then $d_{Z}$ and $d_{Z}^{*}$ are invariant to every reparametrization.

The procedures for constructing pragmatic hypotheses induced by KL and CD also satisfy an additional property given by Theorem 3.

**Theorem** **3.** *Let $Z_{m}=\left(Z_{1},\ldots ,Z_{m}\right)$, where $Z_{i}$’s are i.i.d. $F_{\theta }$ and ${\left(F_{\theta }\right)}_{\theta \in \Theta }$ is identifiable [40,41]. Also, let $KL_{m}$ and $CD_{m}$ be the dissimilarities calculated using $Z_{m}$. If Pg is logically coherent, then, for every $\Theta_{0}\subseteq \Theta$ and ϵ>0,*

*(i)*  ${\left(Pg(\Theta_{0},KL_{m},\epsilon )\right)}_{m\geq 1}$ and ${\left(Pg(\Theta_{0},CD_{m},\epsilon )\right)}_{m\geq 1}$ are non-increasing sequences of sets
*(ii)*  $Pg\left(\Theta_{0},KL_{m},\epsilon \right)\overset{m\to \infty }{\to }\Theta_{0}$ and $Pg\left(\Theta_{0},CD_{m},\epsilon \right)\overset{m\to \infty }{\to }\Theta_{0}$.

Theorem 3 states that the sequence of pragmatic hypotheses for $\Theta_{0}$ induced by $d_{Z_{m}}$ is non-increasing if the dissimilarity is evaluated by either KL or CD. The greater the number of observable quantities $Z_{m}$, the easier it is to distinguish two parameter values $\theta_{0}$ and $\theta^{*}$, and therefore the smaller the amount of parameters that are taken as close to $\theta_{0}$. Also, as the sample size goes to infinity, the pragmatic hypothesis associated to $\Theta_{0}$ converges to to $\Theta_{0}$. In other words, for each $\theta_{0}\in \Theta_{0}$, no other parameter value can predict infinitely many observable quantities with a precision sufficiently close to that of $\theta_{0}$.

## 4. Applications

In the following, pragmatic hypotheses for standard statistical problems are derived. Coscrato et al. [42] provide additional examples and methods for obtaining pragmatic hypotheses.

**Example** **6** (Gaussian with unknown variance.)**.** *Consider the setting from Example 5, but with $\sigma^{2}$ unknown and $0<\sigma^{2}\leq M^{2}$. In this case, the parameter is $\theta =(\mu ,\sigma^{2})$. Consider the composite hypothesis $H_{0}:\left\{\mu_{0}\right\}\times \left(0,M^{2}\right]$, which is often written as $H_{0}:\mu =\mu_{0}$. In this case, let $\theta_{0}=\left(\mu_{0},\sigma_{0}^{2}\right)$ and $\Theta_{0}=\left\{\mu_{0}\right\}\times \left(0,M^{2}\right]$. Proceeding as in Example 5, it follows that*

$$\begin{array}{ccc} Pg(\left\{\theta_{0}\right\},BP_{Z},\epsilon )\; & =\left[\mu_{0}-\epsilon \sigma_{0},\mu_{0}+\epsilon \sigma_{0}\right]\times \left(0,M^{2}\right] & \\ Pg(\Theta_{0},BP_{Z},\epsilon )\; & =\left[\mu_{0}-\epsilon M,\mu_{0}+\epsilon M\right]\times \left(0,M^{2}\right] & Theorem\;1 \end{array}$$

*The rectangular shape of these pragmatic hypotheses seems to be unreasonable, as, for instance, whether a point $\left(\mu ,\sigma^{2}\right)$ is close to $\left(\mu_{0},\sigma_{0}^{2}\right)$ does not depend on $\sigma_{0}^{2}$. This is a consequence of the choice of δ in Example 5.*

**Figure 4:**
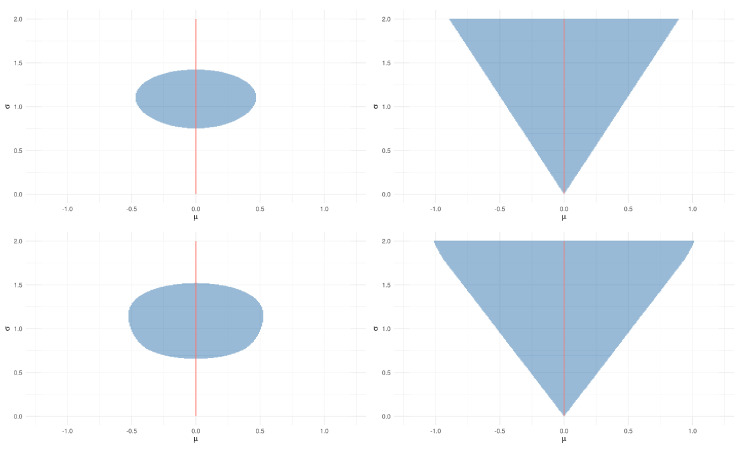
Pragmatic hypotheses in Example 6 for $H_{0}:\mu =0$ with KL (upper), CD (lower), ϵ=0.1, and $M^{2}=2$. $H_{0}$ is represented by a red line in all figures.

Figure 4 presents the pragmatic hypotheses for $H_{0}:\mu =0,\sigma^{2}=1$ and $H_{0}:\mu =0$ when ϵ=0.1 and $M^{2}=2$, and using the KL and CD dissimilarities. Contrary to BP, the hypotheses obtained from these dissimilarities do not have a rectangular shape. In particular, the triangular shape of the pragmatic hypotheses for $H_{0}:\mu =0$ is such that the closer $\sigma^{2}$ is to 0, the smaller the range of values for μ that are included in the pragmatic hypothesis. This behavior might be desirable, as when $\sigma^{2}$ is small, there is little uncertainty about the value of Z, and consequently a narrow interval of values of μ can predict Z with precision ϵ.

**Figure 5:**
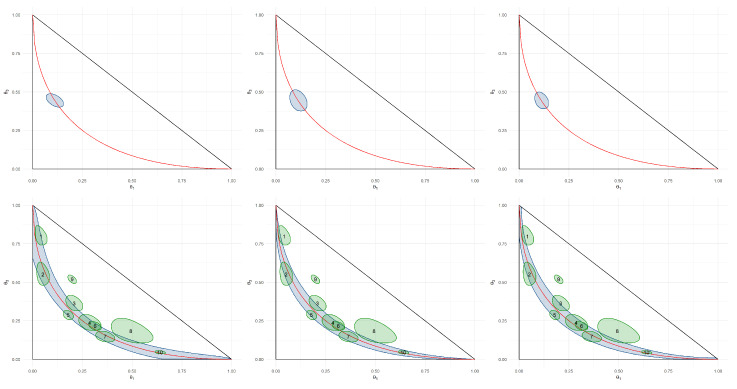
Pragmatic hypotheses obtained for the HW equilibrium, depicted in red, using m=20, ϵ=0.1 for BP and CD and ϵ=0.01 for KL. The blue regions indicate the pragmatic hypothesis for HW and $p=\frac{1}{3}$ (top) and for HW (bottom). The lower, middle, and right panels were obtained, respectively, with BP, KL, and CD. The green regions in the right panels represents 80% HPD regions for the genotype distribution of each of the eight groups collected by Brentani et al. [44] and two simulated datasets.

**Example** **7** (Hardy–Weinberg equilibrium)**.** *Let Z∼Multinomial(m,θ), where $\theta =(\theta_{1},\theta_{2},\theta_{3})$, $\theta_{i}\geq 0$, and $\sum_{i=1}^{3}\theta_{i}=1$. The Hardy–Weinberg (HW) hypothesis [43], $H_{0}$, which is depicted in the red curve in Figure 5 satisfies*

$$\begin{array}{ccc} H_{0}:\theta \in \Theta_{0}, & \; & \Theta_{0}=\left\{\left(p^{2},2p\left(1-p\right),{\left(1-p\right)}^{2}\right):0\leq p\leq 1\right\} \end{array}$$

*If $\theta_{0}^{p}=\left(p^{2},2p\left(1-p\right),{\left(1-p\right)}^{2}\right)$, $\delta_{Z}\left(z\right)={E[∥Z-z∥}_{2}^{2}\left|\theta =\theta_{0}^{p}\right]$ and $g\left(x\right)=\sqrt{x}$, then it follows from Example 2 that*

$$\begin{array}{cc} {BP}_{Z}\left(\theta_{0}^{p},\theta^{*}\right)\; & ={\left(m\times \frac{{\left(\theta_{1}-p^{2}\right)}^{2}+{\left(\theta_{2}-2p\left(1-p\right)\right)}^{2}+{\left(\theta_{3}-{\left(1-p\right)}^{2}\right)}^{2}}{p^{2}\left(1-p^{2}\right)+2p\left(1-p\right)\left(1-2p\left(1-p\right)\right)+{\left(1-p\right)}^{2}\left(1-{\left(1-p\right)}^{2}\right)}\right)}^{0.5} \end{array}$$

*The pragmatic hypotheses that are obtained using KL, BP, and CD for the HW hypothesis are depicted in Figure 5. The choice between BP or KL and CD has a large impact over the shape of the pragmatic hypotheses. Although, for BP, the width of the pragmatic hypothesis is approximately uniform along the HW curve, the width of the pragmatic hypotheses obtained using KL and CD is smaller towards the edges of the HW curve. This behavior could be expected, as towards the edges of the HW curve, Z has the smallest variability. The figure also depicts the challenge in calibrating KL. Although the pragmatic hypotheses for BP and CD have similar sizes when using ϵ=0.1, this result was obtained for KL while using ϵ=0.01.*

The pragmatic hypotheses in Figure 5 are further tested using data from Brentani et al. [44], which is presented in Table 2. This study had the goal of verifying association between the APOE-ϵ4 gene and Alzheimer disease. The lower panels of Figure 5 present the 80% HPD regions for the distribution of this gene in each of the eight groups observed in the study. Additionally, they present two simulated datasets, 9 and 10. Groups 9 and 10 were generated by populations that were, respectively, not under and under the HW equilibrium. Group 9 and 10 fall, respectively, outside and inside of the pragmatic hypothesis.

**Example** **8** (Bioequivalence)**.** *Assume that $Z=\left(X,Y\right)∼N(\left(\mu_{1},\mu_{2}\right),\sigma^{2}I_{2})$, with σ known. We derive the pragmatic hypothesis for $H_{0}:\mu_{1}=\mu_{2}$, that is, for $\left\{\left(\mu_{1},\mu_{2}\right)\in R^{2}:\mu_{1}=\mu_{2}\right\}$. Such a test might be used in a bioequivalence study, where X and Y are the concentrations of an active ingredient in a generic (test) drug medication and in the brand name (reference) medication [45], respectively. As $H_{0}$ is composite, it helps to derive the pragmatic hypothesis of its constituents.*

In order to do so, let $\theta_{0}=\left(\mu_{0},\mu_{0}\right)$, $\mu_{0}\in R$, $\theta^{*}=\left(\mu_{1}^{*},\mu_{2}^{*}\right)$, and $H^{\theta_{0}}:\theta =\theta_{0}$. If $\delta_{Z,\theta^{*}}\left(z\right)=E\left[{\left(X-z_{1}\right)}^{2}+{\left(Y-z_{2}\right)}^{2}|\theta =\theta^{*}\right]$ and $g\left(x\right)=\sqrt{x}$, then

$$\begin{array}{cc} {BP}_{Z}\left(\theta_{0},\theta^{*}\right)\; & =\sqrt{\frac{{\left(\mu_{1}^{*}-\mu_{0}\right)}^{2}+{\left(\mu_{2}^{*}-\mu_{0}\right)}^{2}}{2\sigma^{2}}} \end{array}$$

**Figure 6:**
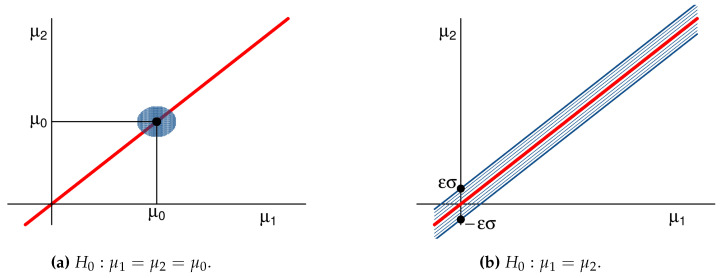
Pragmatic hypotheses using BP in Example 8 when *σ* is known.

Hence, $Pg\left(\left\{\theta_{0}\right\},BP_{Z},\epsilon \right)=\left\{\left(\mu_{1}^{*},\mu_{2}^{*}\right):{\left(\mu_{1}^{*}-\mu_{0}\right)}^{2}+{\left(\mu_{2}^{*}-\mu_{0}\right)}^{2}\leq 2\epsilon^{2}\sigma^{2}\right\}$, which is a circle with center $\left(\mu_{0},\mu_{0}\right)$ and radius $\sqrt{2}\epsilon \sigma$, as depicted on the left panel of Figure 6. In this case, the pragmatic hypothesis is the Tier 1 Equivalence Test hypothesis suggested by the US Food and Drug Administration [45]. The pragmatic hypothesis for $H_{0}:\mu_{1}=\mu_{2}$ is obtained by taking the union of the pragmatic hypotheses associated to its constituents, as illustrated in the right panel of Figure 6. Specifically,

$$\begin{array}{c} Pg\left(H_{0},BP_{Z},\epsilon \right)=\left\{\left(\mu_{1}^{*},\mu_{2}^{*}\right):\left|\mu_{2}^{*}-\mu_{1}^{*}\right|\leq \epsilon \sigma \right\} \end{array}$$

*The pragmatic hypothesis for $H_{0}$ using KL is obtained similarly. Note that*

$$\begin{array}{c} {KL}_{Z}\left(\theta_{0},\theta^{*}\right)=0.5{BP}_{Z}^{2}\left(\theta_{0},\theta^{*}\right) \end{array}$$

Therefore, $Pg\left(\left\{\theta_{0}\right\},KL_{Z},\epsilon \right)=\left\{\left(\mu_{1}^{*},\mu_{2}^{*}\right):{\left(\mu_{1}^{*}-\mu_{0}\right)}^{2}+{\left(\mu_{2}^{*}-\mu_{0}\right)}^{2}\leq 2\epsilon \sigma^{2}\right\}$ and

$$\begin{array}{cc} Pg(H_{0},KL_{Z},\epsilon )\; & =Pg(H_{0},KL_{Z},0.5\epsilon^{2}) \end{array}$$

*The pragmatic hypothesis for $H_{0}$ that is obtained using CD has no analytic expression. However, by observing that $N\left(\mu ,\sigma^{2}\right)=\mu +\sigma N\left(0,1\right)$, it is possible to show that there exists a monotonically increasing function, h:R⟶R, such that*

$$\begin{array}{cc} Pg(H_{0},CD_{Z},0.5\epsilon^{2})\; & =\left\{\left(\mu_{1}^{*},\mu_{2}^{*}\right):\left|\mu_{2}^{*}-\mu_{1}^{*}\right|\leq h\left(\epsilon \right)\sigma \right\} \end{array}$$

That is, the pragmatic hypothesis associated to $H_{0}$ have the same shape as in the right panel of Figure 6. They differ solely on how many standard deviations correspond to the width of the pragmatic hypothesis.

## 5. Final Remarks

The spiral structure studied in the work by the authors of [10] can be used to describe scientific evolution. However, in order for the analogy to be complete, it is necessary to indicate what types of scientific theories or hypotheses are effectively tested in the acceptance vertex of the hexagon of oppositions. We defend that these are pragmatic hypotheses, which are sufficiently precise for the end-user of the theory.

In order to make this statement formal, we introduce three methods for constructing a pragmatic hypothesis associated to a precise hypothesis. These methods are based on three predictive dissimilarity functions: KL, BP and CD. Each of these methods have different advantages. For instance, the scale of BP and CD is more interpretable than KL, making it easier to determine whether the former are large or small. On the other hand, BP relies on the definition of more functions than KL and CD, such as $\delta_{Z,\theta_{0}}\left(z\right)$ in Definition 2. If these function are chosen inadequately, then the shape of the resultant pragmatic hypothesis might be counterintuitive or meaningless. Finally, CD often does not have an analytic expression. It relies on numerical integration over the sample space, which can be taxing in high dimensions.

The applications at Section 4 present adequate choices of metrics and bounds (defining pragmatic hypotheses) for some given theoretical and experimental setups. Nevertheless, the authors did not propose a general or automated recipe for making these choices, nor do they think this to be a feasible goal. In future research, the authors intend to explore a variety of application cases, some using historical data of important experiments, and discuss possible choices of metrics and bounds for each case. The authors hope that, in time, the accumulation of such examples will provide useful guidelines for the good use of methods developed in this paper, in the same way that the statistical literature provides useful guidelines for choosing good statistical models for practical applications.

## Figures and Tables

**Table 1:** Evolution of orbital astronomy and chemical affinity.

Vertex	Orbital Astronomy	Chemical Affinity
$I_{1}$- Enthesis/ $A_{1}$- Thesis	Ptolemaic/Copernican cycles and epicycles	Geoffroy affinity table and highest rank substitution
$U_{1}$- Analysis	Circular or oval orbits?	Ordinal or numeric affinity?
$E_{1}$- Antithesis	Non-circular orbits	Non-ordinal affinity
$O_{2}$- Apothesis /Prosthesis	Elliptic planetary orbits, focal centering of sun	Integer affinity values, for arithmetic recombination
$Y_{2}$- Synthesis	Kepler laws!	Morveau rules and tables!
$I_{2}$- Enthesis $A_{2}$- Thesis	Vortex physics theories, Keplerian astronomy	Affinity + stoichiometry substitution reactions
$U_{2}$- Analysis	Tangential or radial forces?	Total or partial reaction?
$E_{2}$- Antithesis	Non-tangential forces	Non-total substitutions
$O_{3}$- Apothesis/Prosthesis	Radial attraction forces, inverse square of distance	Reversible reactions, equilibrium conditions
$Y_{3}$- Synthesis	Newton laws!	Mass-Action kinetics!
$I_{3}$- Enthesis/ $A_{3}$- Thesis	Newtonian mechanics & variational equivalents	Thermodynamic theories for reaction networks

**Table 2:** Genotype counts for the eight groups in Brentani et al. [44]. Also, the decision of the GFBST agnostic hypothesis test [2] for testing in each group the pragmatic Hardy–Weinberg equilibrium hypothesis with m=20. The decisions are the same for KL, BP, and CD.

	AA	AD	DD	Decision
1	4	18	94	Agnostic
2	6	53	74	Accept
3	57	118	100	Agnostic
4	58	97	48	Agnostic
5	120	361	194	Agnostic
6	206	309	142	Accept
7	110	148	44	Accept
8	34	22	12	Agnostic
9	198	282	520	Reject
10	641	314	45	Accept

## Appendix A. Proofs

**Proof** **of** **Lemma** **1.** Let $\theta \in Pg(\Theta_{0},KL_{Z},8\epsilon^{2})$. It follows from Theorem 1 that there exist $\theta_{0}\in \Theta_{0}$, such that $KL_{Z}\left(\theta_{0},\theta \right)\leq 0.5\epsilon^{2}$. Conclude from Pinsker’s inequality that $CD_{Z}\left(\theta_{0},\theta \right)\leq \sqrt{8^{-1}KL_{Z}\left(\theta_{0},\theta \right)}=\epsilon$, that is, $\theta \in Pg(\Theta_{0},CD_{Z},\epsilon )$. □

**Proof** **of** **Theorem** **1.** Let Pg be logically coherent. Pick an arbitrary $\theta_{0}\in \Theta_{0}$ and note that, if $R\left(x\right)\equiv Pg(\left\{\theta_{0}\right\})$, then $\phi_{Pg(\left\{\theta_{0}\right\})}^{R}\left(x\right)=0$. Since Pg is logically coherent, conclude that $\phi_{Pg(\Theta_{0})}^{R}\left(x\right)\equiv 0$, that is, $Pg\left(\left\{\theta_{0}\right\}\right)\subseteq Pg\left(\Theta_{0}\right)$. Since $\theta_{0}\in \Theta_{0}$ was arbitrary, conclude that

(A1) $$\begin{array}{cc} \underset{\theta_{0}\in \Theta_{0}}{⋃}Pg\left(\left\{\theta_{0}\right\}\right)\; & \subseteq Pg(\Theta_{0}) \end{array}$$

Next, let $R\left(x\right)\equiv {⋂}_{\theta_{0}\in \Theta_{0}}Pg{\left(\left\{\theta_{0}\right\}\right)}^{c}$. For every $\theta_{0}\in \Theta_{0}$, $\phi_{Pg(\left\{\theta_{0}\right\})}^{R}\left(x\right)=1$. Since Pg is logically coherent, $\phi_{Pg(\Theta_{0})}^{R}\equiv 1$, that is, $Pg\left(\Theta_{0}\right)\subseteq R^{c}\equiv {⋃}_{\theta_{0}\in \Theta_{0}}Pg\left(\left\{\theta_{0}\right\}\right)$. Conclude that

(A2) $$\begin{array}{cc} Pg(\Theta_{0}) & \subseteq \underset{\theta_{0}\in \Theta_{0}}{⋃}Pg\left(\left\{\theta_{0}\right\}\right) \end{array}$$

It follows from Equations (A1) and (A2) that $Pg\left(\Theta_{0}\right)={⋃}_{\theta_{0}\in \Theta_{0}}Pg\left(\left\{\theta_{0}\right\}\right)$. It also follows from direct calculation that, if $Pg\left(\Theta_{0}\right)={⋃}_{\theta_{0}\in \Theta_{0}}Pg\left(\left\{\theta_{0}\right\}\right)$, then Pg is logically coherent. □

**Proof** **of** **Theorem** **2.** Let $\Theta_{0}\subseteq \Theta$

$$\begin{array}{cc} Pg(f\left[\Theta_{0}\right],d_{Z}^{*},\epsilon )\; & =\{\theta^{*}\in \Theta^{*}:\exists \theta_{0}^{*}\in f\left[\Theta_{0}\right]\;s.t.\;d_{Z}^{*}\left(\theta^{*},\theta_{0}^{*}\right)\leq \epsilon \}\\ \; & =\{\theta^{*}\in \Theta^{*}:\exists \theta_{0}^{*}\in f\left[\Theta_{0}\right]\;s.t.\;d_{Z}\left(f^{-1}\left(\theta^{*}\right),f^{-1}\left(\theta_{0}^{*}\right)\right)\leq \epsilon \}\\ \; & =f\left[\{\theta \in \Theta :\exists \theta_{0}\in \Theta_{0}\;s.t.\;d_{Z}\left(\theta ,\theta_{0}\right)\leq \epsilon \}\right]\\ \; & =f\left[Pg(\Theta_{0},d_{Z},\epsilon )\right] \end{array}$$

 □

**Proof** **of** **Theorem** **3.** Since the $Z_{i}$’s are i.i.d., ${\mathrm{KL}}_{m}\left(\theta_{0},\theta^{*}\right)=m{\mathrm{KL}}_{Z_{1}}\left(\theta_{0},\theta^{*}\right)$. It follows that

$$\begin{array}{cc} Pg(\Theta_{0},KL_{m},\epsilon )\; & =\underset{\theta_{0}\in \Theta_{0}}{⋃}Pg\left(\left\{\theta_{0}\right\},{\mathrm{KL}}_{m},\epsilon \right)\\ \; & =\underset{\theta_{0}\in \Theta_{0}}{⋃}Pg\left(\left\{\theta_{0}\right\},m{\mathrm{KL}}_{Z_{1}},\epsilon \right)=\underset{\theta_{0}\in \Theta_{0}}{⋃}\left\{\theta^{*}\in \Theta :KL_{Z_{1}}\left(\theta_{0},\theta^{*}\right)\leq m^{-1}\epsilon \right\} \end{array}$$

Thus, ${\left(Pg(\Theta_{0},KL_{m},\epsilon )\right)}_{m\geq 1}$ is a non-increasing sequence of sets. It follows that

$$\begin{array}{cc} \lim_{m\to \infty }Pg\left(\Theta_{0},KL_{m},\epsilon \right)\; & =\underset{m\geq 1}{⋂}\underset{\theta_{0}\in \Theta_{0}}{⋃}\left\{\theta^{*}\in \Theta :{\mathrm{KL}}_{Z_{1}}\left(\theta_{0},\theta^{*}\right)\leq m^{-1}\epsilon \right\}\\ \; & =\underset{\theta_{0}\in \Theta_{0}}{⋃}\underset{m\geq 1}{⋂}\left\{\theta^{*}\in \Theta :{\mathrm{KL}}_{Z_{1}}\left(\theta_{0},\theta^{*}\right)\leq m^{-1}\epsilon \right\}\\ \; & =\underset{\theta_{0}\in \Theta_{0}}{⋃}\left\{\theta^{*}\in \Theta :{\mathrm{KL}}_{Z_{1}}\left(\theta_{0},\theta^{*}\right)=0\right\}\\ \; & =\underset{\theta_{0}\in \Theta_{0}}{⋃}\left\{\theta_{0}\right\}=\Theta_{0} \end{array}$$

where the next-to-last equality follows from the assumption that ${\left(F_{\theta }\right)}_{\theta \in \Theta }$ is identifiable. The proofs for the CD divergence follows from the fact that $\mathrm{TV}\left(P_{\theta_{0}},P_{\theta^{*}}\right)\leq \sqrt{\mathrm{KL}(P_{\theta_{0}},P_{\theta^{*}})}$. □
